# Effects of tai chi, ba duan jin, and walking on the mental health status of urban older people living alone: the mediating role of social participation and the moderating role of the exercise environment

**DOI:** 10.3389/fpubh.2024.1294019

**Published:** 2024-02-08

**Authors:** Baoyuan Wu, Guoyan Xiong, Peng Zhang, Xiujie Ma

**Affiliations:** ^1^School of Wushu, Chengdu Sports University, Chengdu, China; ^2^College of Physical Education and Sports, Beijing Normal University, Beijing, China; ^3^Chinese Guoshu Academy, Chengdu Sports University, Chengdu, China

**Keywords:** tai chi, ba duan jin, walking, urban older people living alone, social participation, exercise environment, mental health

## Abstract

With the global trend of aging, lacking of interpersonal communication and spiritual support and companionship have had a great impact on the mental health of older people living alone. This study examines the multifaceted impacts of engaging in tai chi, ba duan jin, and walking on the mental wellbeing of older people residing alone in urban areas. Additionally, this research aims to explore the association between tai chi, ba duan jin, and walking, and the mental health status of urban older people living alone, by considering the mediating influence of social participation and the moderating influence of the exercise environment. To do so, 1,027 older people living alone in six Chinese cities were investigated using the Physical Activity Rating Scale (PARS-3), the Geriatric Health Questionnaire (GHQ-12), the Social Participation Indicator System Scale, and the Exercise Environment Scale. SPSS 25.0 was utilized for conducting mathematical statistical analysis, specifically for doing linear regression analysis. Additionally, AMOS was employed to develop the study model. We found that a significant negative correlation between tai chi, ba duan jin, and walking and mental health status; among these, tai chi had the greatest impact on the mental health status of urban older people living alone. Social participation mediated the relationship between tai chi, ba duan jin, walking, and mental health status, and the exercise environment had a moderating effect on this relationship. The findings of this study indicate that tai chi, ba duan jin, and walking have a positive impact on the mental health of urban older people living alone, which can be influenced by the mediating efficacy of social participation and the moderating effect of the exercise environment.

## 1 Introduction

The seventh national census predicted that by the end of 2021, 18.9% of China’s population would be over 60 and 14.2% over 65 (1); which marked the beginning of the “silver wave” era in China. At the same time, the family unit is getting smaller. The family unit is growing towards nuclearization and downsizing, and so is the number of older people living alone and empty nesters (2). Older people who live alone are widowed, separated, or have other long-term living reasons and do not have children or other companions nearby, moreover, they are characterized by restricted behavior, poor perceptual skills, low social interaction behavior, and dependency (3). The unique lifestyle and intergenerational interactions of older adults living alone, as opposed to those living with their spouses, children, or grandkids, make them more vulnerable to unpleasant feelings (4). All of these are originated from the family incompleteness, lacking of communication with loved ones, spiritual satisfaction, lack of emotional support, and, over time, loneliness, isolation, and other difficulties overcoming negative emotions. As people age, all of these may be end up with depression, loneliness, and other mental diseases. As a result, older people’s mental health has become a major concern for society.

Socialization has been shown to be important in achieving lifespan and mental health in older persons, and keeping positive relationships with society increases the likelihood of embracing a happy old age (5). Physical engagement has been recommended as early as the 1990s as an activity that enhances an individual’s interpersonal network and has social qualities as they strengthen themselves (6). Physical exercise has now been identified as a driver of physical and mental maintenance, prevention of premature illness, and healthy longevity in older persons (7). Weakened exercise capacity and physical age, decrease the stride speed and horizontal span, poor flexibility, and the physical strength, which prevent the older people to participate in strenuous physical activity. This makes it impossible for the majority of older people to participate in strenuous physical activities (8). In this case, most older people will prioritize sports with low intensity, soft movements and low flexibility requirements. Besides, due to the influence of the traditional Chinese cultural environment, traditional Chinese healthcare sports are highly favored by middle-aged and older people. Among them, tai chi and ba duan jin, as one of the representatives of traditional Chinese healthcare sports programs, have soft and slow as well as continuous and connected movements. They require that “qi is induced by intention, used to move the body, internal qi is generated in the dan tian.” Additionally, the principle of qi and blood circulation of yin and yang and exchange of reality and emptiness regulates the balance of qi and blood in all body areas and promotes older people’s physical and mental health (9). Through a three-month trial, Chan et al. discovered that tai chi can improve the social participation and mental health of isolated older people (10). Another intervention trial study of ba duan jin found that ba duan jin training, combined with cognitive behavioral therapy (CBT), significantly improved the levels of loneliness and depression of homebound older adults as well as their physical and mental health (11). Walking is also an exercise for older people, and it is a low-cost and low-injury strategy to stay healthy that has been shown to improve their mental health (12) and reduce negative feelings (13). Therefore, three exercise programs—tai chi, ba duan jin and walking which older people living alone can participate in on a daily basis, were selected for use in this study to explore the relationship with mental health.

Substantial research has been conducted on the impact of physical exercise participation on older adults’ mental health; however, some gaps remain. For example, active participation in physical exercise by older adults is conducive for the improvement of their social network (14), and higher social participation can provide older adults with more social support and reduce the likelihood of depression, which improves their mental health (15). Nevertheless, few studies have examined the intrinsic links between tai chi, ba duan jin, walking, social participation, and the mental health of older people living alone. Secondly, in the process of older adults’ exercise, the safety of the surrounding exercise environment, rationality of planning, and landscape pleasantness can meet the requirements of older people with regard to the exercise environment. In addition, safe and comfortable sports venues also enable older adults to achieve real relaxation of the body and mind and better experience the beauty of the movement (16). Hence, the exercise environment is a contributing factor to the impression of physical activity in older people. Nevertheless, the existing study has not shown a definitive correlation between the exercise setting and the impact of tai chi, ba duan jin, slow walking, and the mental well-being of elderly individuals who reside alone. Finally, fewer studies have focused on urban older adults living compared to those that have sampled older people or empty nesters, and study sample sizes were also modest.

With the changing family demographic structure in China, the number of older people living alone is increasing, and how to address their unpleasant psychological difficulties has become a pressing issue. As a result, this study chooses social participation as the mediating variable and exercise environment as the moderating variables to investigate the effects of tai chi, ba duan jin, and walking on the psychological health of urban older adults living alone. In addition, it attempts to clarify the relationship between tai chi, ba duan jin, and walking, social participation, exercise environment, and psychological health, as well as its internal mechanism, to provide a reference point for achieving healthy aging in this demographic group.

## 2 Development of hypotheses

### 2.1 Relationship between tai chi, ba duan jin, and walking and mental health

Since its inception, pertinent research has demonstrated that the traditional Chinese national sports and health regimens of tai chi and ba duan jin have a considerable positive impact on the mental and physical wellbeing of their practitioners. Long-term practice of tai chi has been found to be beneficial for mental health, as evidenced by the reduction of psychological issues such as anxiety, depression, and mood disorders in older adults (17–19). Li et al. (20) conducted a randomized controlled trial and found that older practitioners of tai chi reported better levels of life satisfaction, positive emotions, and wellbeing, and lower levels of psychological distress, depression, and negative emotions. Practicing ba duan jin for an extended period of time greatly improves the physical function, walking ability, anxiety, and balance of older persons, and it also improves quality of life and lowers pain and falls (21). Exercise in the ba duan jin style helps to develop and increase mental health as well as to calm the body and mind and control emotions (22). In addition, walking is simple and easy to perform, and the amount of activity can be controlled and altered by the individual, it is independent of environmental, equipment, and other factors. Continuous walking exercise can improve the endocrine and hormonal indexes of the older people’s body, reduce the psychology of frustration, and improve depression and mental health (23–25). Consequently, tai chi, ba duan jin, and walking were chosen as exercise programs for this study. Based on the preceding analysis, we propose the following hypothesis:

*Hypothesis I*: Tai chi, ba duan jin, and walking all have different negative correlations with the mental health status of urban older people living alone.

### 2.2 The mediation effects of social participation

Social participation is a vital means for older people to integrate into society and adapt to social change as well as for a relationship between individual behavior and society (26). Active social participation can improve the lives of older people, enabling them to better integrate into the collective environment of the community and to gain greater social support (27), and allow them to stay connected to society and encourage active aging (28). The relationship between tai chi and social participation has now been explored by some scholars. Studies have shown that when performing a tai chi group exercise, the benefits to the practitioners’ physical, emotional, and social functioning are significant (29); In addition, older adults who did not engage in any social activities had their social networks strengthened and their psychosocial wellbeing improved after practicing tai chi with their peers’ assistance (30). Further, participation in tai chi practice stabilizes older adults’ social activeness and reduces the risk of loneliness and social isolation (31). It has also been found that both ba duan jin and slow walking lead to the participants’ improved socialization and the participants’ enhanced social participation (32, 33). As a result, tai chi, ba duan jin and walking are effective approaches to promote social interaction among older persons. Studies have demonstrated that encouraging formal social participation not only reduces depressive symptoms and enhances quality of life, but it also prevents mental health decline (34). In contrast, older adults who rarely or never participate in social activities have higher levels of psychological distress (35), whereas older adults with high levels of social participation are significantly less likely to suffer from mental illness than those with low levels of social participation (36). Increasing social participation can therefore be used to improve the mental health of older people and prevent the onset of chronic diseases in old age (37). Previous research has confirmed that tai chi, ba duan jin and walking influence older people’ social participation and that social participation can influence their mental health. Consequently, can tai chi, ba duan jin, and walking indirectly influence the mental health status of urban older people living alone through social participation? We thus propose the following hypothesis:

*Hypothesis II*: Social participation mediates the relationship between tai chi, ba duan jin, and walking and mental health status.

### 2.3 The moderating effects of the exercise environment

Numerous studies have demonstrated the impact of the exercise environment on practitioners’ mental health. The physical and emotional experiences that exercisers gain from it vary depending on the type of environment in which they practice, which means that the psychological consequences of various exercise environments may also differ. The perceived esthetics of the exercise environment and the facilities’ convenience and accessibility increase the likelihood that people will be physically active (38). In addition, the size of sports equipment and amenities may influence people’s physical exercise behavior (39). In addition, safer walking paths and easy access to facilities affect older people’s active participation in physical activity (40). The exercise environment influences physical exercise as well as its effects on the practitioner’s perceived mood and wellbeing, and even the same behavior may have opposite effects depending on the exercise environment (41). Furthermore, physical exercise levels are influenced by the exercise environment (42), and the more the outdoor and indoor physical activity facilities (e.g., with walking paths/trails, outdoor tennis courts, gardens, etc.), the greater the number of older adults who do physical exercise, the greater the frequency and duration of exercise, and the higher its level (43). As a result, the exercise environment has a significant role in affecting activity levels in older persons (44). When participants exercised in a better environment with better facilities, tai chi, ba duan jin, and walking had a stronger influence on their mental health condition. In other words, the exercise environment may strengthen the link between tai chi, ba duan jin, and walking and mental wellness. As a result, the following hypothesis was advanced:

*Hypothesis III*: The exercise environment moderates the relationship between tai chi, ba duan jin, walking and mental health status.

**Figure 1:**
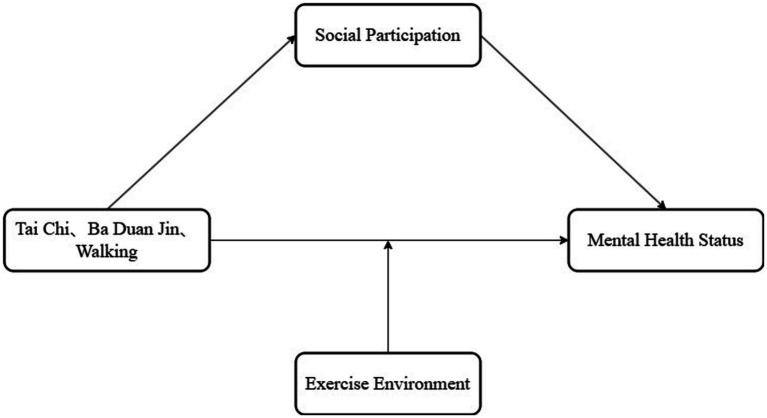
A hypothesized model of the relationship between tai chi, ba duan jin, and walking and mental health status.

In summary, this study built a research model of the mediating and moderating mechanisms of tai chi, ba duan jin, and walking with the mental health status of urban older people living alone, using mental health status as the dependent variable; tai chi, ba duan jin, and walking as the independent variables; social participation as the mediator variable, and exercise environment as the moderating variable (Figure 1). It provides implications for future studies on tai chi, ba duan jin, walking, and the mental health of older people living alone to promote their mental health.

## 3 Materials and methods

### 3.1 Study design and participants

**Figure 2:**
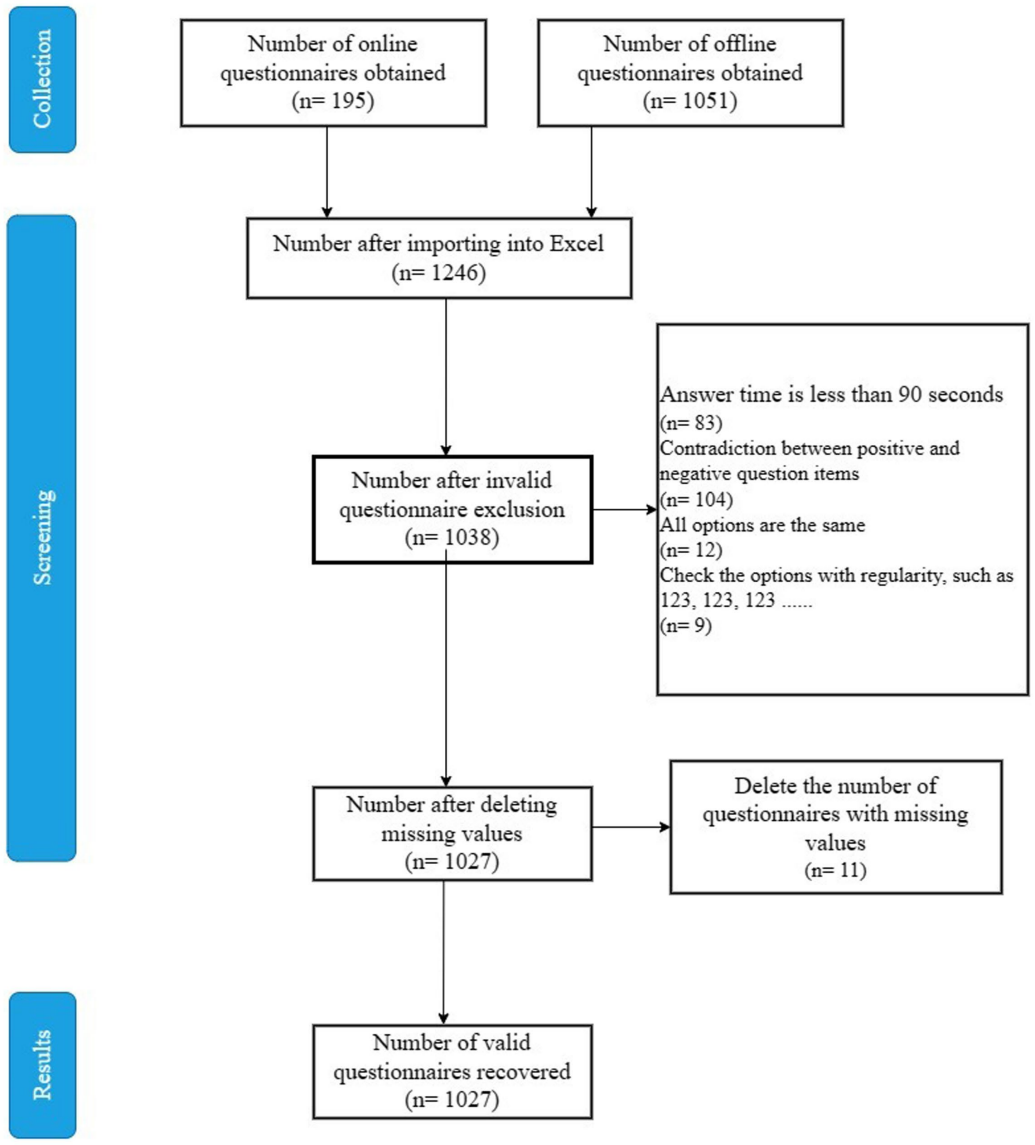
Steps in the screening process for research samples.

The number of senior people (60 and older) in Chengdu City was expected to reach 3.208 million by the end of 2021, accounting for 20.61% of all households. The proportion of older persons in homes aged 70 and up to the total geriatric population climbed to 51.81% (45), demonstrating that the city is already transforming into an aging society. As a result, Chengdu City was selected as the research site, and the findings have promising implications for understanding the mental health and healthy aging of olderly people living alone. In this study, we examined the tai chi, ba duan jin, walking, and mental health of older citizens living alone in six metropolitan areas outside of large cities: Xindu District, Pixian County, Wenjiang District, Shuangliu District, Gaoxin District, and Longquanyi District. We adopted random sampling to ensure the rationality of sample distribution, the number of questionnaires in each urban region was essentially limited to around 200. The inclusion and exclusion criteria had to be defined as we considered the relationship between tai chi, ba duan jin, walking, and the mental health status of urban older people living alone. Criteria for inclusion: (1) individuals 60 years of age or older; (2) Chengdu permanent residents; (3) older people who fit the description of living alone, meaning they are childless or separated and have lived alone for at least a year; (4) informed consent and voluntary participation; and (5) clear consciousness, the ability to read and verbally express oneself, and the ability to interact with the investigators are required. Exclusion criteria were (1) questionnaires that took less than 90 s to complete and (2) responses that were identical, duplicated, or invalid. Figure 2 depicts the screening procedure for the study sample.

The questionnaire was administered on-site and complemented by an electronic questionnaire on the Questionnaire Star website, which took around 3–5 min to complete. Two to three researchers contacted administrators of community or street offices to conduct household surveys, as well as visiting areas where the older people are active, and they used Questionnaire Star to distribute the questionnaires to the designated groups. Prior ethical approval was obtained from the Chengdu Institute of Physical Education. Before the participants completed the questionnaire, the researcher explained the study’s original goal, as well as how the research data would be used and what the associated risks were. Then, they presented the informed consent form to the participants and asked them to sign it. The researcher would answer any questions that the participant did not understand and, if necessary, provide an oral explanation in the participant’s dialect. After completing the survey, participants were given red packets or gifts as a token of appreciation. Finally, we recovered 1,246 questionnaires, including 1,051 offline questionnaires and 195 online questionnaires, with 1,027 valid questionnaires retrieved at an effective recovery rate of 82.4%.

### 3.2 Measurement tool design and reliability testing

#### 3.2.1 Exercise scale

Measurement of tai chi, ba duan jin, and walking exercise among urban older people living alone was conducted using the Physical Activity Rating Scale (PARS-3) revised by Liang et al. (46, 47), which is divided into three dimensions: tai chi, ba duan jin, and walking. A five-point scale from 1 to 5 was used to examine the amount of exercise in terms of three dimensions: intensity, time, and frequency of participation in physical activity. Exercise amount = intensity × time × frequency, intensity, and frequency from 1 to 5 grades, respectively, scored 1–5 points; moreover, time from 1 to 5 grades, respectively, scored 0–4 points, with the highest score being 100 points and the lowest being 0 points. The higher the total score measured, the greater the amount of exercise was. The activity rating scale was as follows: ≤ 19 = low exercise; 20–42 = moderate exercise; ≥43 = heavy exercise. The Cronbach’s α coefficients of these three scales in this study were 0.809, 0.825, and 0.806, respectively.

#### 3.2.2 Mental health status scale

Mental health status was measured using the Geriatric Health Questionnaire (GHQ-12), a self-assessment screening tool that has been successfully used in Chinese samples (48, 49). The GHQ-12 is a unidimensional scale with 12 items on a 0–1 scale; each entry presents four options, with the first two responses scored as 0 and the last two scored as 1 point, with the total score ranging from 0 to 12 and a cut-off value of 3. A total score of 3 indicates a proclivity to develop a psychiatric disorder, while a score of less than 3 indicates normalcy. Higher total GHQ-12 scores are associated with lower levels of mental health. In this investigation, the Cronbach’s α coefficient for this scale was 0.947.

#### 3.2.3 Social participation scale

The social participation index system constructed by Xiu-Ping Wei was used to assess the social participation level of the older people living alone (50, 51). It contains two dimensions of cultural organization activities and personal activities in family affairs and scored on a 5-point Likert scale, with “not participating” scored as 1, “not monthly, but sometimes” scored as 2, “not weekly, but at least once a month” scored as 3, “not daily, but at least once a month” scored as 4, and “not daily, but at least once a week” scored as 5; higher scores indicate higher levels of participation. In this study, the first 25% of the overall score of social participation was classified as high, the middle 50% as medium, and the final 25% as low. In this investigation, the Cronbach’s α coefficient for this scale was 0.867.

#### 3.2.4 Exercise environment scale

The exercise environment was measured using the revised exercise environment scale by Choe et al. (52), which is divided into four dimensions: walkability, safety, exercise equipment, and exercise center. The total score ranges from 0 to 4, and a higher score indicates a higher level of exercise environment. In this study, the first 25% of the overall score of exercise environment was classified as high, the middle 50% as medium, and the final 25% as low. The Cronbach’s α coefficient for the scale in this study was 0.677.

#### 3.2.5 Control variables

Since the mental health of urban older people living alone can be affected by personal and social factors, we used gender, age, education, monthly income, and marital status as control variables to reduce the risk of statistical bias.

#### 3.2.6 Statistical analysis

First, we evaluated the valid questionnaire data with SPSS26.0. We employed AMOS 24.0 to validate the model, which was estimated using the great likelihood approach, Further, we examined the structural validity of the scale. To establish cluster validity, we conducted a confirmatory factor analysis (CFA) and utilized average variance extracted (AVE) and combination reliability (CR) to further examine the scale’s reliability and validity.

Second, we adopted Pearson’s correlation coefficient and other methods to analyze the linear associations of tai chi and ba duan jin with walking, social participation, and mental health status of urban older people living alone. Then, we validated the model using AMOS24.0 to verify the mediating role of social participation between tai chi, ba duan jin, walking, and mental health status. Moreover, we employed the Bootstrap method to test whether a mediating effect of social participation exists between tai chi, ba duan jin, walking, and mental health status of urban older people living alone. Currently, the Bootstrap technique is a popular way to test the mediating impact. This method entails repeating sampling from the original sample and determining whether or not the coefficient of the mediating effect is significant using a 95% confidence interval (53).

Finally, we utilized linear regression to examine the role of exercise environment in the association between tai chi, ba duan jin, and walking and the mental health status of urban older people living alone. In this study, we performed the three-step test of Hierarchical Moderated Regression (HMR) analysis and used the interaction terms of the variables to test for moderating effects. In more detail, we conducted the empirical test as follows, with SPSS 26.0 utilizd to statistically analyze tai chi, ba duan jin, and walking. Correlation analysis was utiliszd for first hypothesis testing based on testing for common method bias, followed by moderated model testing using linear regression analysis. Step by step, this model was separated into three linear regression models. As shown in Table 1, model 1 (M1) was fitted first, with gender, age, education, monthly income, and marital status as control variables and tai chi, ba duan jin, and walking scores as independent variables for regression fitting. Model 2 then added a moderating variable (exercise environment) on top of model 1. Model 3 finally added an interaction term (product term of the independent and the moderating variables) on top of model 2.

**Table 1:** Moderated effects test results (*n* = 1,027).

	Variable	Model 1			Model 2			Model 3		
		B	t	*p*	B	t	*p*	B	t	*p*
TC	Constant	2.327	15.536	0.000**	2.349	15.825	0.000**	2.329	16.011	0.000**
	TC	−0.35	−14.143	0.000**	−0.348	−14.23	0.000**	−0.28	−10.554	0.000**
	EE				−0.292	−5.215	0.000**	−0.377	−6.635	0.001**
	TC*EE							−0.396	−5.981	0.000**
	R ^2^	0.169			0.191			0.219		
	Adjusted R ^2^	0.162			0.183			0.21		
	*F*-value	*F* (9, 1,017) = 23.062, *p* = 0.000			F (10, 1,016) = 24.011, *p* = 0.000			F (11, 1,015) = 25.827, *p* = 0.000		
	△R ^2^	0.169			0.022			0.028		
	△F value	F (9, 1,017) = 23.062, *p* = 0.000			*F* (1, 1,016) = 27.200, *p* = 0.000			*F* (1, 1,015) = 35.769, *p* = 0.000		
BDJ	Constant	2.386	15.59	0.000**	2.409	16.021	0.000**	2.412	16.196	0.000**
	BDJ	−0.317	−12.382	0.000**	−0.327	−12.989	0.000**	−0.285	−10.741	0.000**
	EE				−0.354	−6.227	0.000**	−0.401	−7.008	0.000**
	BDJ*EE							−0.309	−4.544	0.000**
	R^2^	0.136			0.168			0.185		
	Adjusted R^2^	0.129			0.16			0.176		
	F-value	F (9, 1,017) = 17.838, *p* = 0.000			F (10,1,016) = 20.528, *p* = 0.000			F (11, 1,015) = 20.900, *p* = 0.000		
	△R ^2^	0.136			0.032			0.017		
	△F value	F (9, 1,017) = 17.838, *p* = 0.000			F (1, 1,016) = 38.780, *p* = 0.000			F (1, 1,015) = 20.650, *p* = 0.000		
W	Constant	2.301	15.15	0.000**	2.313	15.375	0.000**	2.302	15.521	0.000**
	W	−0.326	−12.86	0.000**	−0.319	−12.694	0.000**	−0.239	−8.318	0.000**
	EE				−0.263	−4.618	0.000**	−0.343	−5.902	0.004**
	W*EE							−0.393	−5.481	0.000**
	R ^2^	0.145			0.163			0.187		
	Adjusted R^2^	0.138			0.154			0.178		
	F-value	F (9, 1,017) = 19.189, *p* = 0.000			F (10, 1,016) = 19.747, *p* = 0.000			F (11, 1,015) = 21.196, *p* = 0.000		
	△R ^2^	0.145			0.018			0.024		
	△F value	F (9, 1,017) = 19.189, *p* = 0.000			F (1, 1,016) = 21.322, *p* = 0.000			F (1, 1,015) = 30.040, *p* = 0.000		

Dependent variable, mental health status; * *p* < 0.05; ** *p* < 0.01; TC, Tai Chi; BDJ, Ba Duan Jin; W, Walking; SP, Social Participation; EE, Exercise Environment; MHS, Mental Health Status.

#### 3.2.7 Validity testing

**Figure 3:**
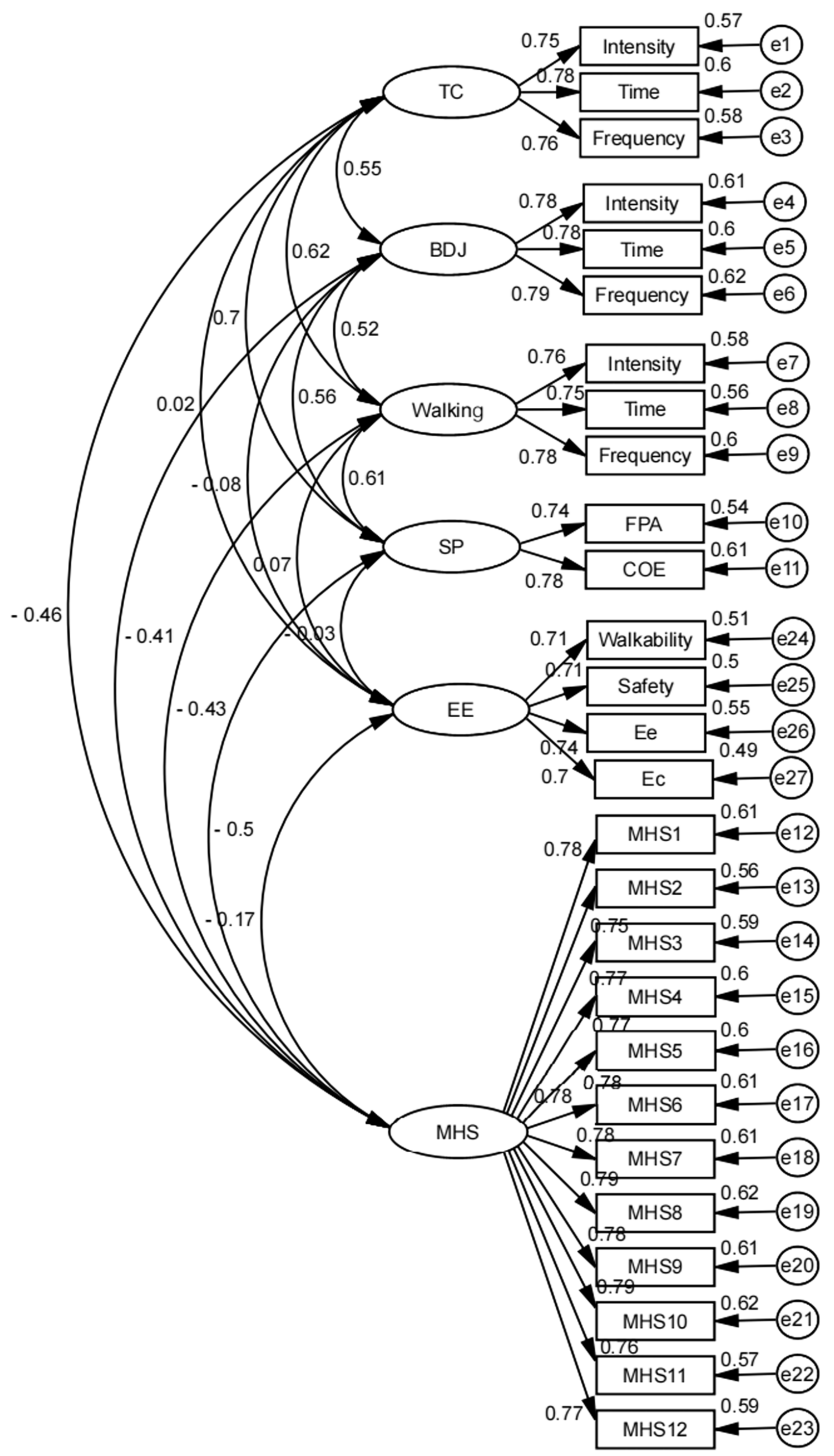
Validated factor analysis model diagram (TC, Tai Chi; BDJ, Ba Duan Jin; SP, Social Participation; EE, Exercise environment; MHS, Mental Health Status; FPA, Family Personal Activities; COE, Cultural Organization Events; Ee, Exercise equipment; Ec, Exercise center).

First, as shown in Figure 3, the absolute values of the standardized loading coefficients are all more than 0.6 and exhibit significance when each measurement relationship is examined; this shows that the measurement relationship is sound. Furthermore, as shown in Table 2, all AVE values for each factor are greater than 0.5, and all CR values for each factor are greater than 0.7; this implies that the data of this analysis have good aggregation (convergence) validity and thus good construct validity and consistency. Again, most of the model-fitting indicators of the validated factor analysis results in Table 3, *χ*^2^/ df < 3, GFI > 0.9, RMSEA < 0.10, RMR < 0.05, CFI > 0.9, NFI > 0.9, TLI > 0.9, reach the standard, showing that the model fits well. As a result, the questionnaire in this research has a high level of internal reliability and validity.

**Table 2:** Questionnaire reliability and validity tests.

Variable	CR	AVE
TC	0.809	0.585
BDJ	0.825	0.611
Walking	0.806	0.581
SP	0.731	0.576
EE	0.807	0.511
MHS	0.947	0.6

TC, Tai Chi; BDJ, Ba Duan Jin; SP, Social Participation; EE, Exercise environment; MHS, Mental Health Status.

**Table 3:** Questionnaire model fitting indicators.

	*χ* ^2^	*df*	χ^2^/*df*	GFI	RMSEA	RMR	CFI	NFI	TLI
Model	480.774	309	1.556	0.968	0.023	0.019	0.988	0.968	0.987

GFI, goodness-of-fit index; RMSEA, root mean square error of approximation; RMR, root mean square error; CFI, comparative fit index; NFI, normed fit index; TLI, Tucker-Lewis index.

## 4 Results

### 4.1 Sample situation analysis

This study collected a total of 1,027 valid questionnaires, as indicated in Table 4. The participants included 443 men (43.14%) and 584 women (56.86%), of whom the majority were between the ages of 60 and 64 (37.97%); most of them had an elementary school education or less (46.93%), were widowed, and had a monthly income ranging from 3,000 to 4,000 yuan.

**Table 4:** Demographic characteristics of the sample.

Variable	Frequency	Percentage
*Gender*		
Men	443	43.14
Women	584	56.86
*Age*		
60–64	390	37.97
65–69	248	24.15
70–74	191	18.6
75–79	105	10.22
≥80	93	9.06
*Education level*		
Primary and below	482	46.93
Middle School	348	33.89
High school and above	197	19.18
*Marital status*		
Unmarried	11	1.07
Separated	381	37.1
Divorced	184	17.92
Widowed	451	43.91
*Income*		
<3,000	219	21.32
3,000–4,000	423	41.19
4,000–5,000	290	28.24
>5,000	95	9.25
*Tai Chi*		
Mild exercise	458	44.6
Moderate exercise	277	26.97
Intense exercise	292	28.43
*Ba Duan Jin*		
Mild exercise	507	49.37
Moderate exercise	260	25.32
Intense exercise	260	25.32
*Walking*		
Mild exercise	462	44.99
Moderate exercise	274	26.68
Intense exercise	291	28.33
*Mental health status*		
No mental problems	390	37.97
Possible mental problems	637	62.03
*Social participation*		
Low level of social participation	272	26.48
Medium LEVEL of social participation	483	47.03
High social participation	272	26.48
*Exercise environment*		
Low exercise environment level	253	24.63
High exercise environment level	774	75.37

In addition, older people living alone participated in tai chi (44.6%), ba duan jin (49.37%), and walking (44.99%) with less exercise; more older people living alone were found to have possible psychological problems (62.03%); 47.03% had a moderate level of social participation, and 75.37% were in a high level of exercise environment. Although the sample data cannot explicitly demonstrate the association between tai chi, ba duan jin, and walking and the mental health status of older adults living alone, they help readers and researchers thoroughly comprehend the sample’s characteristics.

### 4.2 Descriptive statistics and correlation analysis

Based on the descriptive statistics, Table 5 shows the mean, standard deviation, correlation coefficient of the main variables. A significant negative correlation existed between tai chi, ba duan jin, walking, and mental health status (TC: *r* = −0.405, *p* < 0.01; BDJ: *r* = −0.361, *p* < 0.01; Walking: *r* = −0.373, *p* < 0.01). A significant positive correlation existed between tai chi, ba duan jin, walking, and social participation (TC: *r* = 0.529, *p* < 0.01; BDJ: *r* = 0.436, *p* < 0.01; Walking: *r* = 0.480, *p* < 0.01), and a significant negative correlation was found between social participation and mental health status (*r* = −0.420, *p* < 0.01). Thus, H1 and H2 were initially supported.

**Table 5:** Descriptive statistics and correlation of variables.

Variable	M	S.D.	TC	BDJ	Walking	SP	EE	MHS
TC	31.094	25.009	0.765					
BDJ	29.321	24.841	0.451**	0.782				
Walking	31.109	24.712	0.497**	0.425**	0.762			
SP	26.848	5.847	0.529**	0.436**	0.480**	0.759		
EE	2.283	1.575	0.017	−0.068*	0.057	−0.027	0.715	
MHS	5.078	4.57	−0.405**	−0.361**	−0.373**	−0.420**	−0.151**	0.775

* *p* < 0.05; ** *p* < 0.01. M, Mean; S.D., Standard Deviation; TC, Tai Chi; BDJ, Ba Duan Jin; SP, Social Participation; EE, Exercise Environment; MHS, Mental Health Status.

### 4.3 Analysis of mediation effects

**Figure 4:**
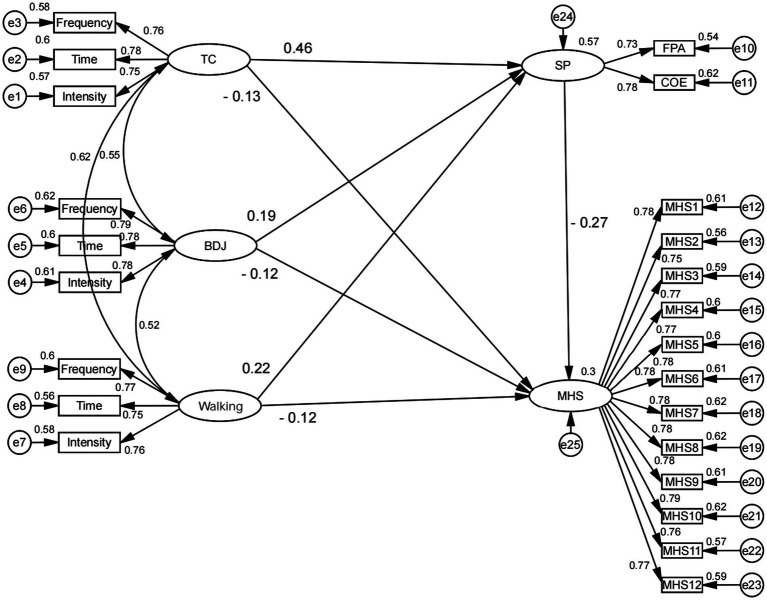
Structural equation modeling on the mediating role of social participation between tai chi, ba duan jin, walking, and mental health status (TC, Tai Chi; BDJ, Ba Duan Jin; SP, Social Participation; EE, Exercise environment; MHS, Mental Health Status; FPA, Family Personal Activities; COE, Cultural Organization Events; Ee, Exercise equipment; Ec, Exercise center).

Initially, based on the mediated effect model fitting indices presented in Table 6 (*χ*^2^/df < 3, GFI > 0.9, RMSEA <0.10, RMR < 0.05, CFI > 0.9, NFI > 0.9, TLI > 0.9), most of the model-fitting indices met the required threshold, indicating a well-fitted mediated effect model affecting the mental health status of older people living alone. Figure 4 illustrates the significant negative correlation between tai chi, ba duan jin, and walking on the mental health status of older people living alone in the city. The path coefficients of tai chi, ba duan jin, and walking → mental health status (TC: *β* = −0.13; BDJ: *β* = −0.12; Walking: *β* = −0.12) are significant, indicating a direct effect of tai chi, ba duan jin, and walking on mental health status (Figure 4). The significant negative correlation suggests that tai chi exercise has a significant positive effect on the mental health condition of older adults living alone. H1 is valid because the question item indicates that tai chi from 1 to 5 indicates that the intensity ranges from weak to strong, and the mental health condition from 1 to 4 indicates that the mental health condition changes from good to bad. Furthermore, a mediating effect of social participation was observed between tai chi, ba duan jin, walking, and mental health status, as indicated by the path coefficients of tai chi, ba duan jin, walking → social participation (TC: *β* = 0.46; BDJ: *β* = 0.19; Walking: *β* = 0.22) and social participation → mental health condition (*β* = −0.27). We examined H2 in a preliminary manner.

**Table 6:** Model fit indices for the mediating effects of tai chi, ba duan jin, walking, social participation, and mental health status.

	*χ* ^2^	df	χ2/df	GFI	RMSEA	RMR	CFI	NFI	TLI
Model	393.054	220	1.787	0.969	0.028	0.022	0.987	0.971	0.985

GFI, goodness-of-fit index; RMSEA, root mean square error of approximation; RMR, root mean square error; CFI, comparative fit index; NFI, normed fit index; TLI, Tucker-Lewis index.

The number of repeated samplings of the original sample must be at least 1,000 times in the Bootstrap mediated effects test (54), and if the Bootstrap mediated effects test result shows that the Bootstrap test CI does not contain a value of 0, the indirect effect is significant (55). In this study, we estimated the mediating effect Bootstrap95% CI using a sample of 5,000 times to investigate whether social participation mediated the association between tai chi, ba duan jin, walking, and mental health status. The findings are displayed in Table 7. The indirect effects of tai chi, ba duan jin, walking → social participation → mental health status were − 0.123, −0.051, and − 0.058, respectively, with Z-values of −3.324, −3.000, and − 1.871, respectively. Moreover, the Bootstrap 95% CI generated by this pathway did not contain 0: the 95% CI using the bias-corrected Bootstrap method yielded lower bounds of −0.212, −0.093, and − 0.136 and upper bounds of −0.06, −0.023, and − 0.011, respectively; and the 95% CI using the bias-corrected nonparametric percentile Bootstrap method yielded lower bounds of −0.203, −0.086, and − 0.129, and the upper limits of −0.055, −0.019, and − 0.008, respectively. This indicates that the mediating effect of social participation between tai chi, ba duan jin, walking, and mental health status was significant, with walking having the highest mediating effect; thus, H2 is valid. The direct effects of tai chi, ba duan jin, and walking → mental health status were − 0.132, −0.125, and − 0.119, respectively, and the z-values were − 2.164, −3.378, and − 2.245, respectively. The Bootstrap 95% confidence intervals generated by this pathway did not contain 0 for the direct indirect effects, and the total effects of tai chi, ba duan jin, and walking → mental health status were − 0.255, −0.255, −0.255, and − 0.255, respectively, −0.255, −0.176, −0.177, with Z-values of −5.426, −4.889, −3.933, respectively, and the Bootstrap 95% confidence intervals generated by this pathway did not contain 0. This indicates that the direct and total effects of tai chi, ba duan jin, and walking on mental health status were significant. In conclusion, since both the direct and indirect effects were significant, the three mediating effects of tai chi, ba duan jin, and walking were all partially mediated. Furthermore, the sequence of the effect sizes of tai chi, ba duan jin, and walking on the mental health status of older people living alone in the city is tai chi → walking → ba duan jin by comparing the size of the total effect value of these three activities on mental health status.

**Table 7:** Mediation effect test results.

Path		Estimate	Bootstrapping				SE	Z	STD. estimate
			Bias-corrected		Percentile				
			Lower	Upper	Lower	Upper			
Direct effect	TC → MHS	−0.128	−0.246	−0.007	−0.247	−0.007	0.061	−2.164	−0.132
	BDJ → MHS	−0.119	−0.189	−0.044	−0.19	−0.047	0.037	−3.378	−0.125
	Walking→MHS	−0.115	−0.212	−0.005	−0.212	−0.006	0.053	−2.245	−0.119
Indirect effect	TC → SP → MHS	−0.12	−0.212	−0.06	−0.203	−0.055	0.037	−3.324	−0.123
	BDJ → SP → MHS	−0.049	−0.093	−0.023	−0.086	−0.019	0.017	−3.000	−0.051
	Walking→SP → MHS	−0.057	−0.136	−0.011	−0.129	−0.008	0.031	−1.871	−0.058
Total effect	TC → MHS	−0.248	−0.343	−0.163	−0.34	−0.16	0.047	−5.426	−0.255
	BDJ → MHS	−0.167	−0.236	−0.096	−0.236	−0.097	0.036	−4.889	−0.176
	Walking→MHS	−0.172	−0.259	−0.084	−0.259	−0.083	0.045	−3.933	−0.177

TC, Tai Chi; BDJ, Ba Duan Jin; SP, Social Participation; EE, Exercise Environment; MHS, Mental Health Status.

### 4.4 Analysis of moderation effects

Hypothesis H3 proposes that the higher the level of exercise environment, the higher the degree of influence of tai chi, ba duan jin, and walking on mental health status. For model 1, which aims to investigate the influence of the independent variables (tai chi, ba duan jin, and walking) on the dependent variable (mental health status) when the interference of the moderating variable (exercise environment) is not considered, Table 1 shows that tai chi, ba duan jin, and walking were significant (TC: *t* = 14.143, *p* < 0.05; BDJ: *t* = −12.382, *p* < 0.05; walking: *t* = −12.86, *p* < 0.05) and would thus have a significant effect relationship on mental health status, further validating Hypothesis H1. The test of the interaction between the effects of tai chi, ba duan jin, and walking and the exercise environment on mental health status (M2 & M3) found significant changes in *F* values from M2 to M3, with TC: *F* (10, 1,016) = 24.011, *p* < 0.05 → *F* (11, 1,015) = 25.827, *p* < 0.05; BDJ: F (10, 1,016) = 20.528, *p* < 0.05 → F (11, 1,015) = 20.900, *p* < 0.05; walking: *F* (10, 1,016) = 19.747, *p* = 0.135 → F (11, 1,015) = 21.196, *p* < 0.05. In addition, as shown in Table 1, the interaction terms of tai chi, ba duan jin, and walking with the exercise environment showed significance (TC: *t* = −5.981, *p* < 0.05; BDJ: *t* = −4.544, *p* < 0.05; walking: *t* = −5.481, *p* < 0.05). When affecting mental health status, the magnitude of the moderating variable (exercise environment) was significantly different at different levels.

**Figure 5:**
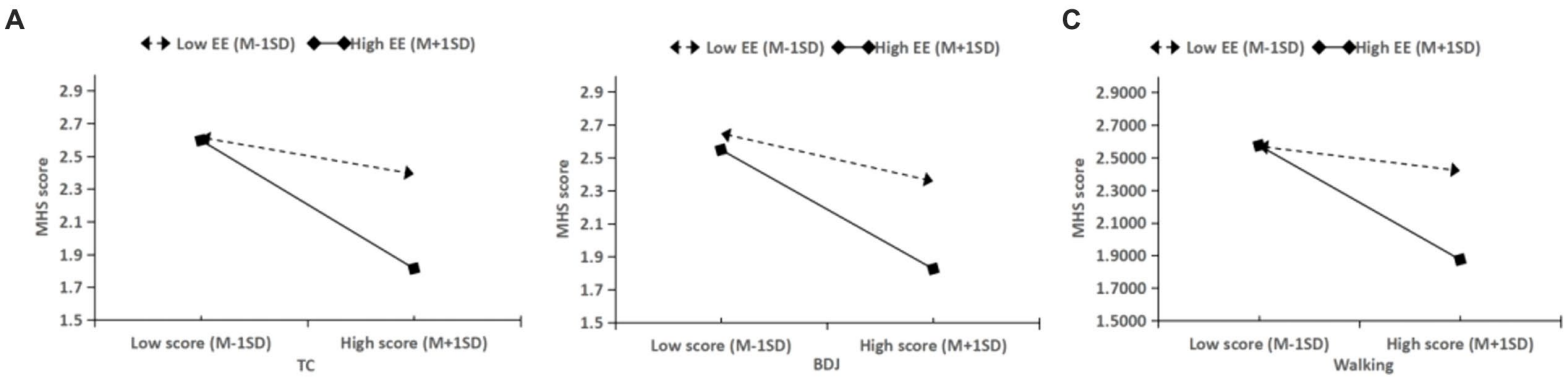
Moderating role of exercise environment between tai chi, ba duan jin, walking and mental health status. **(A)** Simple slope analysis of the moderating effect of exercise environment on the relationship between tai chi and mental health status; **(B)** simple slope analysis of the moderating effect of exercise environment on the relationship between ba duan jin and mental health status; **(C)** simple slope analysis of the moderating effect of exercise environment on the relationship between walking and mental health status.

To clearly present the moderating effect of exercise environment between tai chi, ba duan jin, walking, and mental health status, based on the method suggested by Aiken and West (56), the current study divided the mean (M) plus or minus one standard deviation (SD) of the exercise environment into high and low groupings and plotted the simple slope test graph (Figure 5). The test results revealed that the impacts of tai chi, ba duan jin, and walking on mental health status were stronger in high-exercise environments and weaker in low-exercise environments. This suggests that as the degree of exercise environment increases, the effect of tai chi, ba duan jin, and walking on the psychological health status of urban older people living alone improves, and hypothesis H3 is established.

## 5 Discussion

### 5.1 Tai chi, ba duan jin, walking, and mental health status of urban older adults living alone

This study aims to investigate the association between involvement in tai chi, ba duan jin, and walking and the mental health status of urban older adults living alone in China. According to the findings, most older adults living alone had poor levels of activity and psychological difficulties. Moreover, a substantial negative association was observed between tai chi, ba duan jin, walking, and mental health state, which was confirmed by correlation analysis. The result is consistent with previous findings indicating that the higher the degree of tai chi, ba duan jin, and walking among older people living alone, the lower the risk of psychological illnesses (57–59). The total effect values in Table 7 suggest that tai chi has the greatest impact on the mental health of older adults living alone. This could be because practicing tai chi allows practitioners to meditate and concentrate while avoiding distractions, and the abdominal breathing method utilized in tai chi is also good medicine for stress treatment. Studies have found that tai chi reduces anxiety and depression symptoms in practitioners and improves their mental health (60). A meta-analysis of 37 randomized controlled trials and five quantitatively lenient studies found that tai chi interventions improve various mental health indicators in different populations, including depression, anxiety, general stress management, and exercise self-efficacy (61). Another study discovered that frequent tai chi practice effectively reduces negative emotions (62) and boosts life satisfaction and wellbeing in older adults (20), improving their mental health even further. A clinical study showed that long-term walking and tai chi exercise can help middle-aged and older adults feel better emotionally, with particular benefits for depression (63). Walking has a clear impact on the release of negative emotions in older adults; this can raise hormone levels and make individuals feel less frustrated and less depressed, all of which lower the risk of mental issues (64). Regular practice of ba duan jin can also enhance mood, quality of life, and sleep among older people (65, 66). Regular practice of ba duan jin has been shown, in a randomized controlled experiment, to successfully manage tranquility and pleasantness, reduce anxiety, and improve the physical and mental health of older persons (67). As a result, the positive effects of tai chi, ba duan jin, and walking can also benefit the mental health status of urban older people living alone, confirming the research hypothesis that tai chi, ba duan jin, and walking are all differentially and negatively correlated with their mental health status.

### 5.2 The mediating effect of social participation

The study results confirmed H2, which indicates a significant mediating effect of social participation between tai chi, ba duan jin, walking, and the mental health status of urban older adults living alone. Tai chi interventions among older adults have been shown to increase social support and promote social participation (68), while higher levels of social participation are linked to lower levels of psychological disorders (69). In other words, social participation improves mental health, and engaging in social activities can consistently contribute to an individual’s well-being in later life (70). In addition, among the direct effects of tai chi, ba duan jin, and walking on the mental health status of older adults people living alone, tai chi had the most significant effect, but of the indirect effects, walking produced the highest effect value, followed by ba duan jin. Walking has reduced exercise requirements, including its lower intensity and expense, could be the reason why older persons prefer to take part in such activities (71). Additionally, since older adults living alone spend the majority of their time on their own, the neighborhood recreational spaces such as community centers, shopping centers, and parks that they visit on a daily basis provide opportunities for walking (72), and walking to active places becomes an important physical exercise for them. Furthermore, studies have indicated that older people’ social participation is more likely to be influenced by their ability to participate in physical activities including walking, jogging, and cycling in urban areas (73). According to Sun et al. (74), social participation improves older adults’ mental health in both urban and rural areas. For example, it increases life satisfaction (75), decreases depression (76, 77), improves cognitive health, and lowers cognitive dysfunction in older adults (78). In a similar vein, boosting social participation levels and frequency of social encounters could help older persons with anxiety and depression by improving their mental health and quality of life (79). This viewpoint is further supported by this study’s results. Tai chi, ba duan jin, and walking all affected the level of social participation of urban older adults living alone. Moreover, social participation has an effect on the mental health status of this group, and it also mediates the relationship between tai chi, ba duan jin, and walking and mental health status.

### 5.3 The moderating effect of the exercise environment

The study also sought to determine the exercise environment’s moderating role in the relationship between tai chi, ba duan jin, and walking and mental health status; in other words, the effects of tai chi, ba duan jin, and walking on mental health status were strengthened when the level of the exercise environment was high. This shows that exercising in an area with a high degree of exercise environment can benefit older persons’ mental health condition, which echoes the findings of earlier studies (80, 81). Research has found that with various walking paths in the exercise environment, older adults are more physically active (82), and factors such as accessibility to public spaces, proximity to amenities, the presence of parks near the community, and high levels of safety all positively influence older adults’ physical exercise and health behaviors (83). Furthermore, Kim and Wu et al. indicated that exercising in green surroundings such as urban parks, walking greenways, and recreational equipment is more helpful to the mental health of older persons (84, 85). On one hand, walkable environments with pedestrian-supportive infrastructures, such as wooden benches for resting, safe vehicular access, nearby amenities, and appropriate public transportation, can play a beneficial role in physical exercise, particularly for older adults living alone with limited mobility and functional limitations (86). On the other hand, exercising in high safety, walkability, good access to parks, and neighborhood environments make older adults feel more at ease and relaxed than in less favorable environments; thus, physical exercise in the latter environments has a better effect on older adults’ mental health status (87, 88). This finding provides more evidence for the moderating impact of the exercise environment on the mental health of older adults living alone in urban areas.

### 5.4 Contributions

Firstly, based on earlier research, the impacts of tai chi, ba duan jin and walking on the mental health status of urban older adults living alone were investigated in greater depth by linking social participation, exercise environment, and mental health status. Secondly, this study found and verified the mediating relationship of social participation between tai chi, ba duanjin, and walking and the mental health of urban older adults living alone and provided reference suggestions for improving and enhancing the quality of their support system of social participation and mental health. In addition, the rest of the findings suggest a moderating effect of the exercise environment on the relationship between tai chi, ba duan jin, and walking and the mental health of urban older adults living alone. A better exercise environment has potential benefits of physical exercise, so it is a good choice for the communities to improve their unqualified exercise environments. Ultimately, this study provides directions and references for more mediating and moderating methods to enhance the mental health of urban older people living alone.

### 5.5 Limitations and future research directions

This study had limitations. Firstly, it only discussed three types of exercise— tai chi, ba duan jin, and walking—while disregarding other types of sports. Traditional Chinese sports such as six-character formula, five-animal exercise, yi jin jing, and tai chi sword may be studied in the future. Furthermore, we used the PARS-3 scale to assess the daily participation of older adults living alone in tai chi, ba duan jin, and walking in terms of the three dimensions of exercise intensity, time, and frequency; however, we did not assess other types of physical exercise. The International Physical Activity Questionnaire may be expanded in future research to include additional sports. Secondly, the Exercise Environment Scale employed in the study examined the exercise environments of senior individuals living alone in four dimensions, namely, walkability, safety, exercise equipment, and exercise facilities; however, it did not allow for a comparison of indoor and outdoor exercise environments. Future studies could expand on this basis by comparing indoor and outdoor workout conditions.

Finally, we used a cross-sectional study, which may not be able to infer causal relationships between variables as in other cross-sectional analysis studies. The study of tai chi, ba duan jin, and walking, and the mental health state of older adults living alone can be investigated in future research by a follow-up design and experimental research. In the meantime, the internal processes connecting the mental health condition of older single people and tai chi, ba duan jin, and walking are numerous; additional mediating and moderating variables, such as the community setting, social support, interpersonal relationships, and exercise modality, can be investigated. In addition, although this study set gender, age, education, monthly income, and marital status as control variables, control for covariates is lacking, which is a major limitation of this study. Future research should expand the model even more.

## 6 Conclusion

This study, distinctively, examined the links between tai chi, ba duan jin, walking, social participation, exercise environment, and mental health status in a sample of urban older Chinese people living alone. We observed a negative association between tai chi, ba duan jin, and walking and the mental health status of this group, with tai chi having the most influence. The relationship between mental health status and tai chi, ba duan jin, and walking was mediated by social participation, and the exercise environment could regulate the effects of tai chi, ba duan jin, and walking on mental health status. This study provides evidence to help clarify the association between tai chi, ba duan jin, and walking and the mental health condition of urban older adults living alone, which is essential to prevent mental issues in this group.

This study contends that the intervention role of social participation in the mental health status of urban older people living alone should be addressed, and that frequent tai chi, ba duan jin, and walking exercises can aid in mental health improvement. It is also critical to emphasize the importance of the exercise environment and to provide better exercise environment and conditioned facilities for older adults living alone to increase the attractiveness of the exercise environment to them, improve their level of physical exercise, and prevent the emergence of psychological problems.

## Data availability statement

The original contributions presented in the study are included in the article/supplementary material, further inquiries can be directed to the corresponding author.

## Ethics statement

The studies involving humans were approved by the Chengdu Sport University. The studies were conducted in accordance with the local legislation and institutional requirements. The participants provided their written informed consent to participate in this study.

## Author contributions

BW: Conceptualization, Investigation, Methodology, Project administration, Writing – original draft, Writing – review & editing. GX: Methodology, Writing – original draft, Writing – review & editing. PZ: Methodology, Writing – review & editing. XM: Conceptualization, Data curation, Funding acquisition, Methodology, Project administration, Resources, Software, Validation, Visualization, Writing – original draft, Writing – review & editing.
